# Omega-3 fatty acids eicosapentaenoic acid and docosahexaenoic acid and their mechanisms of action on apolipoprotein B-containing lipoproteins in humans: a review

**DOI:** 10.1186/s12944-017-0541-3

**Published:** 2017-08-10

**Authors:** Jan Oscarsson, Eva Hurt-Camejo

**Affiliations:** AstraZeneca Gothenburg, Pepparedsleden 1, SE-431 83 Mölndal, Sweden

**Keywords:** Eicosapentaenoic acid (EPA), Docosahexaenoic acid (DHA), Very-low-density lipoproteins, Low-density lipoproteins, Chylomicrons, Triglycerides, Lipoprotein lipase, Apolipoprotein B, Apolipoprotein E, Apolipoprotein CIII

## Abstract

**Background:**

Epidemiological and genetic studies suggest that elevated triglyceride (TG)-rich lipoprotein levels in the circulation increase the risk of cardiovascular disease. Prescription formulations of omega-3 fatty acids (OM3FAs), mainly eicosapentaenoic acid (EPA) and docosahexaenoic acid (DHA), reduce plasma TG levels and are approved for the treatment of patients with severe hypertriglyceridemia. Many preclinical studies have investigated the TG-lowering mechanisms of action of OM3FAs, but less is known from clinical studies.

**Methods:**

We conducted a review, using systematic methodology, of studies in humans assessing the mechanisms of action of EPA and DHA on apolipoprotein B-containing lipoproteins, including TG-rich lipoproteins and low-density lipoproteins (LDLs). A systematic search of PubMed retrieved 55 articles, of which 30 were used in the review; 35 additional arrticles were also included.

**Results:**

In humans, dietary DHA is retroconverted to EPA, while production of DHA from EPA is not observed. Dietary DHA is preferentially esterified into TGs, while EPA is more evenly esterified into TGs, cholesterol esters and phospholipids. The preferential esterification of DHA into TGs likely explains the higher turnover of DHA than EPA in plasma. The main effects of both EPA and DHA are decreased fasting and postprandial serum TG levels, through reduction of hepatic very-low-density lipoprotein (VLDL)-TG production. The exact mechanism for reduced VLDL production is not clear but does not include retention of lipids in the liver; rather, increased hepatic fatty acid oxidation is likely. The postprandial reduction in TG levels is caused by increased lipoprotein lipase activity and reduced serum VLDL-TG concentrations, resulting in enhanced chylomicron clearance. Overall, no clear differences between the effects of EPA and DHA on TG levels, or on turnover of TG-rich lipoproteins, have been observed. Effects on LDL are complex and may be influenced by genetics, such as *APOE* genotype.

**Conclusions:**

EPA and DHA diminish fasting circulating TG levels via reduced production of VLDL. The mechanism of reduced VLDL production does not involve hepatic retention of lipids. Lowered postprandial TG levels are also explained by increased chylomicron clearance. Little is known about the specific cellular and biochemical mechanisms underlying the TG-lowering effects of EPA and DHA in humans.

## Background

Triglycerides (TGs) are carried in the circulation as TG-rich lipoprotein particles, mainly in the very-low-density lipoprotein (VLDL) fraction in the fasted state and as VLDL and chylomicrons in the fed state. High plasma TG levels that meet the definition of mild-to-moderate hypertriglyceridemia (2–10 mmol/L [175–885 mg/dL]) strongly enhance the risk of cardiovascular (CV) disease [1, 2]. Adjustment for high-density lipoprotein (HDL)- and non-HDL-cholesterol markedly reduces the association between plasma TGs and CV disease, indicating a role in the enhanced CV disease risk for cholesterol carried in the atherogenic remnant lipoproteins associated with hypertriglyceridemia. However, Mendelian randomization studies indicate a direct causal relationship between TG-rich lipoproteins and coronary heart disease [1, 2]. Severe hypertriglyceridemia, defined as TG levels greater than 10 mmol/L (885 mg/dL), is mainly associated with an increased risk of acute pancreatitis [2, 3]. Hypertriglyceridemia is a common disorder, with polygenic and lifestyle factors as its main etiology; monogenic etiology is much rarer. The prevalence of mild-to-moderate hypertriglyceridemia estimated from the Copenhagen General Population Study was 28%, while the prevalence of severe hypertriglyceridemia was 0.1% [2]. The prevalence of high TG levels (200–<500 mg/dL) was 16.2% and of very high TG levels (≥500 mg/dL) was 1.1% in the US adult population [4].

First-line treatment for patients with hypertriglyceridemia is management of diet (reduced dietary fat, simple carbohydrates and alcohol) and more exercise. Fibrates are recommended as first-line pharmacologic therapy in patients with severe hypertriglyceridemia, while statins are recommended as first-line treatment in patients with mild-to-moderate hypertriglyceridemia [1, 3]. Statins sometimes need to be combined with a fibrate, niacin or omega-3 fatty acids (OM3FAs) to manage mild-to-moderate hypertriglyceridemia [1, 3]. However, the US Food and Drug Administration recently withdrew previous approvals recommending the combination of statins with fibrates or niacin. The decision was prompted by the results of three large trials, which failed to show a reduction in CV events with these therapy combinations [5]. OM3FA treatment on top of statins is well tolerated and effectively lowers levels of VLDL particles and TGs [6, 7]. Prescription-grade OM3FA formulations are now the remaining approved combination therapy with statins for further reduction of plasma TGs in patients with severe hypertriglyceridemia and increased CV risk; the combination of statins with OM3FAs is therefore of great clinical importance. Doses of about 2–4 g of prescription-grade OM3FAs, mainly containing eicosapentaenoic acid (EPA) and docosahexaenoic acid (DHA), reduce plasma TG levels by about 30% and are indicated for prevention of pancreatitis in patients with severe hypertriglyceridemia [1, 3]. There is a lack of evidence that OM3FA treatment reduces CV events in patients with hypertriglyceridemia of any severity, as recently reviewed by Siscovick and colleagues [8]. Two randomized outcome trials, REDUCE-IT (REDUuction of Cardiovascular Events with EPA - Intervention Trial) and STRENGTH (Outcomes Study to Assess STatin Residual Risk Reduction with EpaNova in HiGh CV Risk PatienTs with Hypertriglyceridemia), are ongoing to assess residual risk reduction when adding OM3FA therapy to statins [9, 10]. Aside from effects on TG-rich lipoproteins, OM3FAs may reduce CV risk via other mechanisms such as effects on arrhythmias and inflammation, as reviewed by de Roos and colleagues [11]. However, reduction in atherosclerosis in animal models or humans by administration of OM3FAs has not been convincingly demonstrated [8, 11].

Here, we review the mechanisms of action of the OM3FAs EPA and DHA on production and clearance of apolipoprotein (apo)B-containing lipoproteins, including TG-rich lipoproteins (VLDLs, chylomicrons) and low-density lipoproteins (LDLs). The emphasis is on results obtained in clinical studies. Many studies have demonstrated the TG-rich lipoprotein-lowering effects of OM3FAs, with mechanistic studies focussing on animal models. Less is known about the specific metabolism and effects of the two principal constituents of prescription OM3FA formulations, EPA and DHA, and their mechanisms of action in humans. For further reading, a meta-analysis and systematic review was published on this topic by Wei and Jacobson [12]. Another recommended review is a comprehensive summary of kinetic studies on the effects of OM3FAs on apoB-containing lipoproteins, published by Harris and Bulchandani [13]. Finally, a review that also includes results from mechanism of action studies of OM3FAs on TG metabolism in cultured cells and animal models was published by Davidson [14].

### Search strategy

PubMed was searched to identify articles of relevance to the review, using combined search terms from three categories: (1) ‘DHA/EPA’; (2) ‘outcomes’ (i.e. lipoproteins, TGs, etc.); and (3) ‘mechanism of action’ (Table 1). The terms ‘comparative’ and ‘controlled’ were included among the mechanism of action terms to ensure that clinical studies that focus on the differential effects of DHA and EPA on outcomes (such as TGs and lipoproteins) but do not discuss their mechanism of action were not excluded from the search results. Review articles were excluded and PubMed filters were applied to further limit identified articles to human studies and publications written in English.

**Table 1:** Search terms for the systematic PubMed search

Search string category	Search terms used
(1) DHA/EPA	‘docosahexaenoic acid’ *OR* ‘docosahexaenoic’ *OR* ‘eicosapentaenoic acid’ *OR* ‘eicosapentaenoic’ *OR* ‘icosapentaenoic acid’
(2) Outcomes	‘triglyceride’ *OR* ‘triacylglycerol’ *OR* ‘triacylglycerols’ *OR* ‘triacylglyceride’ *OR* ‘triacylglycerides’ *OR* ‘lipoprotein’ *OR* ‘HDL’ *OR* ‘HDL-C’ *OR* ‘HDLC’ *OR* ‘LDL’ *OR* ‘LDL-C’ *OR* ‘LDLC’ *OR* ‘VLDL’ *OR* ‘VLDL-C’ *OR* ‘VLDLC’ *OR* ‘apolipoprotein’ *OR* ‘postprandial lipids’ *OR* ‘post-prandial lipids’
(3) Mechanism of action	‘production’ *OR* ‘clearance’ *OR* ‘mode of action’ *OR* ‘mechanism of action’ *OR* ‘controlled’ *OR* ‘comparative’
Combined search	(1) *AND* (2) *AND* (3)

*DHA* docosahexaenoic acid, *EPA* eicosapentaenoic acid, *HDL* high-density lipoprotein, *HDLC/HDL-C* high-density lipoprotein cholesterol, *LDL* low-density lipoprotein, *LDLC/LDL-C* low-density lipoprotein cholesterol, *VLDL* very low-density lipoprotein, *VLDLC/VLDL-C* very low-density lipoprotein cholesterol

**Fig. 1:**
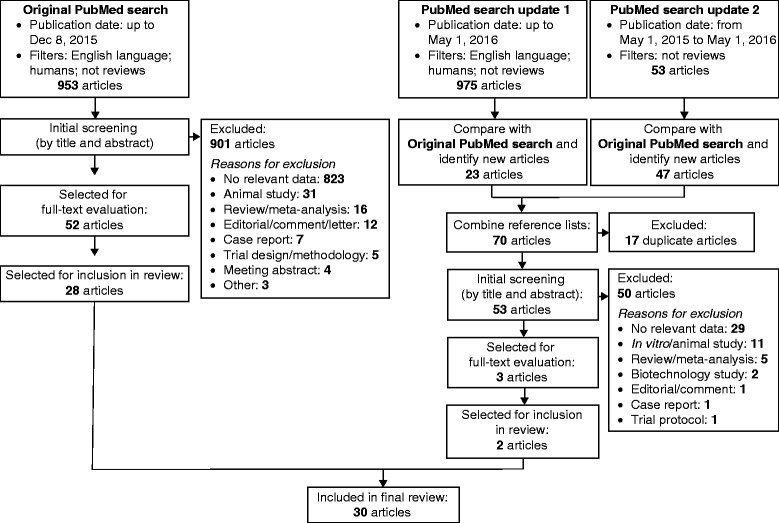
Flow diagram of article screening and evaluation

The systematic search process is summarized in Fig. 1 *.* The original literature search was performed on December 8, 2015 and identified a total of 953 articles for screening, of which 52 were selected for full-text evaluation; 28 of these were used in the review. The most common reasons for excluding articles from the systematic search were either that they did not contain relevant data relating to the effects of DHA and EPA on lipoprotein metabolism, or that they did not report clinical studies. A further literature search process was performed on May 1, 2016 to identify articles published since the original search, using two approaches. First, a new search was performed using the same terms as the original search, applying a publication cut-off date of May 1, 2016; this revealed 23 new articles. Secondly, another new search was performed using the original terms but with a publication date range of May 1, 2015 to May 1, 2016 and without the filter for human studies; this was to ensure the detection of any recently added articles that may have been missing the appropriate Medical Subject Heading (MeSH) terms for human studies. Combining the articles identified using the two update searches and discarding duplicates revealed 53 new articles for screening. Three articles were selected for full-text evaluation, 2 of which were used in the review. Thus, from an overall total of 55 potentially relevant articles identified by the systematic search (52 from the original search and 3 from the update search), 30 were used for the review (Table 2).

**Table 2:** Characteristics of included studies identified by the systematic PubMed search

Study [reference]	Study design	Population, number of participants	Treatment groups (daily dose indicated^a^)	Control	Duration	Major finding(s)
Ågren et al. [27]	Single-blind, controlled	Healthy men, *n =* 59	Control, fish diet, 4 g fish oil (1.33 g EPA + 0.95 g DHA), 1.68 g DHA	Standard diet	15 weeks	DHA reduced plasma TGs
Buckley et al. [23]	Double-blind, placebo-controlled	Normolipidemic adults, *n =* 42	Control, 4.8 g EPA, 4.9 g DHA	Olive oil	4 weeks	TG-lowering more effective with DHA than with EPA
Chan et al. [29]	Double-blind, placebo-controlled	Dyslipidemic, obese men, *n =* 24	Control, 1.8 g EPA + 1.56 g DHA	Corn oil	6 weeks	VLDL-apoB production decreased
Dawson et al. [63]	Double-blind, placebo-controlled	Hypertriglyceridemic men, *n =* 4	Control, 3 g DHA	Olive oil	90 days	Reduced expression of LDL receptor and inflammatory markers in blood cells
Egert et al. [18]	Single-blind, uncontrolled	Normolipidemic men and women, *n =* 74	4.4 g ALA, 2.2 g EPA, 2.3 g DHA	N/A	6 weeks	Serum TGs decreased similarly with DHA and EPA, no effects of DHA or EPA on LDL
Grimsgaard et al. [26]	Double-blind, placebo-controlled	Healthy, non-smoking men, *n =* 234	Control, 3.8 g EPA, 3.6 g DHA	Corn oil	7 weeks	Serum TGs decreased similarly with DHA and EPA, no effects of DHA or EPA on LDL
Hansen et al. [21]	Double-blind, uncontrolled	Healthy, normolipidemic men, *n =* 14	3.8 g EPA, 3.6 g DHA	N/A	5 weeks	DHA numerically reduced postprandial TGs more than EPA
Harris et al. [43]	Single-blind, placebo-controlled	Healthy, normolipidemic adults, *n =* 20, and hypertriglyceridemic adults, *n =* 6	Control, 5 g fish oil (2 g EPA + 1.14 g DHA) Control, 5 g fish oil (2 g EPA + 1.14 g DHA)/70 kg	Olive oil	3 weeks 4 weeks	Plasma (non-heparin-stimulated) LPL activity increased
Homma et al. [45]	Open, uncontrolled	Hypertriglyceridemic men and women, *n =* 15	2.7 g EPA	N/A	12 weeks	VLDL-apoCII and VLDL-apoCIII decreased, small LDL increased
Lindsey et al. [61]	Open, uncontrolled	Healthy, normolipidemic men and women, *n =* 7	3.6 g EPA + 2.9 g DHA	N/A	2 weeks	Larger LDL following active treatment, reduced LDL receptors on HepG2 cells compared with baseline LDL
Mori et al. [22]	Double-blind, placebo-controlled	Hypertriglyceridemic men, *n =* 59	Control, 3.8 g EPA, 3.7 g DHA	Olive oil	6 weeks	Serum TGs decreased similarly with DHA and EPA; DHA, but not EPA, increased LDL
Nenseter et al. [51]	Open, controlled	Normolipidemic men and women, *n =* 23	Control, 5.1 g EPA + DHA	Corn oil	4 months	No difference in uptake of LDL in HepG2 cells between control and active treatment; no effect on LDL size
Nestel et al. [28]	Open, controlled	Healthy, normolipidemic adults, *n =* 5, and hyperlipidemic adults, *n =* 2	Control, fish oil (up to 30% of energy needs)	Safflower oil	2–3.5 weeks	Reduced VLDL-TG and VLDL-apoB production, no change in FFA flux
Nordoy et al. [52]	Double-blind, placebo-controlled	Hyperlipidemic men and women, *n =* 42	Control, 0.9 g EPA + 0.8 g DHA	Corn oil	5 weeks	No effect on non-heparin or post-heparin plasma LPL activity
Nozaki et al. [57]	Open, uncontrolled	Hyperlipidemic men and women, *n* = 14	2.4 g EPA	N/A	6 months	Total cholesterol, TG, LDL-C plasma levels significantly reduced; LDL particle size unchanged; CETP activity significantly reduced
Olano-Martin et al. [5]	Double-blind, cross-over, placebo-controlled	Healthy, normolipidemic men, *n =* 38	Control, EPA 3.3 g, DHA 3.7 g	80:20 palm olein:soy bean mixture	4 weeks	LDL levels increased in *APOE4* carriers on DHA, VLDL2 from *APOE4* carriers inhibited LDL uptake in HepG2 cells
Ouguerram et al. [30]	Open, uncontrolled	Patients with type 2 diabetes mellitus and dyslipidemia, *n =* 5	1080 mg EPA + 720 mg DHA	N/A	8 weeks	VLDL1 production rate decreased and fractional catabolic rate was unchanged
Park and Harris [38]	Double-blind, placebo-controlled	Healthy, normolipidemic men and women, *n =* 33	Control, 3.8 g EPA, 3.8 g DHA (EPA and DHA groups were pooled)	Safflower oil	4 weeks	Chylomicron TG half-lives decreased, pre-heparin LPL activity increased, DHA and EPA were equally effective
Park et al. [39]	Double-blind, placebo-controlled	Healthy, normolipidemic men and women, *n =* 33	Control, 3.8 g EPA, 3.8 g DHA	Safflower oil	4 weeks	DHA but not EPA increased margination volume as an estimate of LPL binding capacity in the fed state
Rambjor et al. [20]	Single-blind, placebo-controlled	Normolipidemic men and women, *n =* 49 (two cross-over studies and one parallel-arm study)	Control, 2.7 g EPA, 2.5 g DHA, 5 g fish oil (2.05 g EPA + 1.15 g DHA)	Olive oil	3 weeks	EPA, but not DHA, decreased TGs, VLDL-C and increased LDL-C
Rudkowska et al. [42]	Open, uncontrolled	Men with PPARα-V162 allele, *n* = 14, matched with men homozygous for PPARα-L162, *n* = 14	Mix of 1.9 g EPA + 1.1 g DHA	N/A	6 weeks	EPA and DHA increase LPL transcription independent of PPARα genetic variation
Sanders et al. [15]	Double-blind, placebo-controlled	Hypertriglyceridemic men, *n =* 21; VLDL kinetics were studied in 5 patients	Control, 15 g fish oil (2.9 g EPA + 1.95 g DHA)	Olive oil and corn oil blend	4 weeks	DHA increased in VLDL-TGs, while EPA increased mainly in VLDL PLs; no effect on VLDL fractional catabolic rate
Schmidt et al. [62]	Open, uncontrolled	Normolipidemic men, *n =* 10, and dyslipidemic men, *n =* 10	Fish oil (1.56 g EPA + 1.14 g DHA)	N/A	12 weeks	Whole blood expression of LDL receptor mRNA decreased in dyslipidemic men
Schwellenbach et al. [25]	Double-blind, uncontrolled	Patients with CAD and hypertriglyceridemia, *n =* 116	1000 mg DHA, 1252 mg DHA + EPA	N/A	8 weeks	A greater proportion of patients receiving DHA achieved a TG level < 150 mg/dL
Tani et al. [53]	Open, controlled	Hypertriglyceridemic men and women, *n =* 144	Control, 1800 mg EPA	No treatment	6 months	No change in LDL-C but LDL size increased
Tatsuno et al. [24]	Double-blind, uncontrolled	Hypertriglyceridemic men and women, *n =* 610	0.9 g EPA + 0.75 g DHA, 1.8 g EPA + 1.5 g DHA, 1.8 g EPA	N/A	12 weeks	TG lowering was similar with EPA and EPA + DHA, no difference in LDL reduction between groups
Vidgren et al. [16]	Open, controlled	Healthy, normolipidemic men, *n =* 59	Control, fish diet (0.38 g EPA + 0.67 g DHA five times weekly), 1.68 g DHA, fish oil (1.33 g EPA + 0.95 g DHA)	Standard diet	14 weeks	DHA was incorporated into PLs and TGs, while EPA was incorporated into PLs and CEs; DHA retroconverted to EPA
Wong et al. [40]	Single-blind, controlled	Obese men and women, *n =* 27	Hypocaloric diet alone or in combination with 1.8 g EPA + 1.56 g DHA	Hypocaloric diet	12 weeks	Compared with weight loss alone, EPA + DHA reduced postprandial TGs and apoB48
Woodman et al. [19]	Double-blind, placebo-controlled	Patients with type 2 diabetes mellitus, *n =* 59	Control, 3.8 g EPA, 3.7 g DHA	Olive oil	6 weeks	Similar effects of DHA and EPA on serum lipids, DHA retroconverted to EPA
Woodman et al. [54]	Double-blind, placebo-controlled	Patients with type 2 diabetes mellitus, *n =* 59	Control, 3.8 g EPA, 3.7 g DHA	Olive oil	6 weeks	DHA increased LDL size to a greater extent than EPA

^a^EPA and DHA doses may be proportions of a larger overall dose of oil/OM3FAs

*ALA* alpha-linolenic acid, *Apo* apolipoprotein, *CE* cholesterol ester, *CAD* coronary artery disease, *DHA* docosahexaenoic acid, *EPA* eicosapentaenoic acid, *FFA* free fatty acid, *LDL* low density lipoprotein, *LDL-C* low-density lipoprotein cholesterol, *LPL* lipoprotein lipase, *N/A* not applicable, *OM3FA* omega-3 fatty acid, *PL* phospholipid, *PPAR* peroxisome proliferator-activated receptor, *VLDL* very-low-density lipoprotein, *VLDL-apo* very-low-density lipoprotein apolipoprotein, *VLDL-C* very-low-density lipoprotein cholesterol, *VLDL-TG* very-low-density lipoprotein triglyceride, *TG* triglyceride

An additional 35 articles not identified in the systematic search were included in the review (including the introduction); these were identified during development of the manuscript (citations in papers identified during the screening process, for example). These additional articles were included in the interests of providing context and completeness, and comprise 8 review articles about OM3FAs, 15 original articles about OM3FAs and lipoproteins, and 12 articles about the importance of different lipoproteins in CV disease or mechanisms of action of lipoproteins of general importance for interpreting the OM3FA studies.

## Results

### Differences in the metabolism of DHA and EPA

After supplementation with fish oil (which contains DHA and EPA esterified to TGs and at lower concentrations than prescription-grade OM3FAs), both phospholipids and TGs in VLDL are enriched in EPA, while TGs, but not phospholipids, in VLDL are enriched in DHA [15]. This observation was corroborated in another study investigating enrichment of DHA and EPA in all lipoproteins. DHA was found mainly in TGs, while EPA, but not DHA, was found in cholesterol esters [16]. The preferential incorporation of DHA into TGs could help to explain why total DHA levels in plasma do not increase to the same extent as EPA levels following fish oil supplementation, because turnover of serum TGs is greater than turnover of phospholipids and cholesterol esters. Moreover, combined treatment with EPA and DHA showed that DHA is more available for beta-oxidation than EPA [17], possibly owing to the enrichment of TGs with DHA.

The metabolism of DHA and EPA has been described in clinical studies, with DHA being retro-converted to EPA and EPA being elongated mainly to docosapentaenoic acid (DPA) [18–22]. However, production of DHA from EPA does not seem to occur. Therefore, treatment with purified DHA results in an increase in both DHA and EPA, while treatment with purified EPA does not generally change DHA levels. The increase in EPA levels following DHA intervention can sometimes exceed the increase in DHA plasma levels [18].

### Differential effects of DHA and EPA on TG-rich lipoproteins

In general, the TG-lowering effects of DHA and EPA are similar in both normotriglyceridemic [18, 23] and hypertriglyceridemic patients [19, 22, 24]. In some studies, TG lowering tended to be more effective with DHA than with EPA supplementation in hypertriglyceridemic [18, 25] and normolipidemic individuals [18, 23, 26], while in one study EPA was more effective than DHA in normolipidemic individuals [20]. In normolipidemic individuals, both DHA and EPA decrease postprandial TG levels and it is not possible to tell from these studies whether one of the fatty acids has a superior efficacy [21, 27]. In summary, there is no clear difference between DHA and EPA with respect to reduction in fasting or postprandial TG levels.

### VLDL production and clearance

A meta-analysis of human kinetic studies showed a consistent reduction in VLDL production rate following supplementation with OM3FAs, while effects on VLDL clearance were less consistent [13]. In a study investigating the effect of 3 weeks of fish oil versus safflower oil supplementation, VLDL-apoB and TG kinetics in normolipidemic and hypertriglyceridemic individuals were determined [28]. VLDL-apoB production rate decreased in all individuals, while fractional clearance rate increased in normal individuals but not in hypertriglyceridemic individuals. Moreover, the results indicated that the reduced VLDL production rate was not caused by diminished flux of fatty acids, pointing towards a hepatic effect of OM3FAs on lipid handling. LDL-cholesterol (LDL-C) levels tended to increase and in some individuals an increased influx of LDL was observed, indicating increased hepatic production of LDL particles [28]. In a study comparing 4 weeks of daily supplementation with either 15 g fish oil or 15 g of a blend of corn and olive oil, the clearance rate for VLDL-TGs was not influenced by the treatment [15]. It was therefore concluded that the reduction in plasma TGs was most likely caused by diminished VLDL production, in line with the study of Nestel et al. [28]. In more recent VLDL kinetic studies, lower doses of purified OM3FAs have been investigated. Chan et al. conducted a study comparing 4 g daily of OM3FAs and corn oil in 24 dyslipidemic men [29]. Treatment with OM3FAs decreased hepatic production of VLDL-apoB by 29% more than corn oil. This effect was accompanied by a 35% decrease in hepatic TG synthesis. Moreover, conversion of VLDL-apoB to intermediate-density lipoprotein (IDL)-apoB, and IDL-apoB to LDL-apoB, increased significantly. The explanation for the enhanced conversion of VLDL to IDL and LDL is likely to be a lower number of VLDL particles per lipoprotein lipase (LPL) enzyme, which leads to a greater proportion of particles being converted into LDL [29].

The effects of OM3FAs on VLDL kinetics in patients with type 2 diabetes mellitus and atherogenic dyslipidemia have also been investigated [30]. This is of particular interest because the dyslipidemia associated with type 2 diabetes is characterized by increased VLDL1 (large VLDL) production, which results in hypertriglyceridemia, small dense LDL particles and low HDL-cholesterol levels, i.e. atherogenic or diabetic dyslipidemia. Five patients with type 2 diabetes and atherogenic dyslipidemia were treated daily for 8 weeks with fish oil containing 1.8 g EPA and DHA [30]. Plasma levels of TG and VLDL (including VLDL1) production decreased, and conversion of VLDL to IDL, and IDL to LDL, increased. The fractional clearance rate of VLDL, IDL or LDL did not change [30]. This study shows that OM3FA supplementation partly corrects for the underlying disorder responsible for the atherogenic dyslipidemia in patients with type 2 diabetes by reducing hepatic production of VLDL1.

#### Possible mechanisms explaining reduced VLDL production

Atherogenic dyslipidemia is associated with insulin resistance. While OM3FA treatment could result in a small improvement in hepatic insulin sensitivity [31], other effects of OM3FAs must be of importance to the decrease in VLDL production resulting from OM3FA treatment. Insulin resistance is associated with increased hepatic fat content. In turn, liver fat content is positively associated with VLDL production [32]. Therefore, reduced liver fat content should lead to reduced hepatic VLDL production. A meta-analysis of human intervention studies showed that OM3FAs decrease liver fat content [33], which could help to explain the reduced VLDL production. Studies of OM3FA treatment of patients with non-alcoholic fatty liver disease have shown that reduction in liver fat content was associated with a change in DHA levels, while fasting plasma TG levels were associated with EPA enrichment [34]. These results indicate that the relationship between liver fat reduction and plasma TG levels is not similar for DHA and EPA. The exact mechanisms taking place in human liver cells that explain reduced liver fat and reduced VLDL production are still speculative. Clinical studies have shown that OM3FA treatment increases fatty acid oxidation, as determined by indirect calorimetry [35, 36]. It is therefore possible that OM3FA treatment reduces VLDL production via reduced substrate availability, by increasing fatty acid oxidation, which would reduce liver TG content and substrate availability for VLDL production.

In summary, the major mechanism that explains the reduced fasting serum TGs following OM3FA treatment is reduced VLDL production, including a reduced number and size of VLDL particles. Reduced VLDL secretion enhances the rate of conversion of VLDL particles to IDL and LDL particles.

### Postprandial lipids and chylomicron production and removal

Several studies have investigated the effect of OM3FA supplementation on postprandial lipoprotein levels and metabolism. A study of the acute and chronic effects of 25 days’ OM3FA supplementation (30% of the total fat from fish oil or 3.5 g OM3FA per 1000 kcal) compared with test meals with mainly saturated fatty acids or mainly omega-6 fatty acids (OM6FAs) on postprandial lipoprotein metabolism was performed in eight normolipidemic individuals [37]. It showed that both acute and chronic supplementation with OM3FAs reduced plasma levels of chylomicrons and postprandial TG levels to a greater extent than saturated fatty acid- or OM6FA-enriched meals. However, the mechanisms for the reduced chylomicron levels associated with OM3FA supplementation were not investigated in this study.

The effects of OM3FA treatment on chylomicron clearance have been studied using different methods and the results are consistent with an increased clearance of chylomicrons being the major mechanism that explains reduced postprandial lipid levels [38, 39]. Thirty-three healthy individuals were treated in a randomized, double-blind, placebo-controlled study with either DHA or EPA ethyl esters for 4 weeks, and the effects were compared with those for safflower oil [38]. Both EPA and DHA treatment significantly increased removal of TGs in chylomicrons in the fed state but not in the fasted state. This finding indicates that OM3FA treatment increases the capacity for TG removal, but this increase in clearance capacity is mainly of importance when chylomicron levels are increased. Pre-heparin LPL activity increased following both DHA and EPA treatment [38], indicating that increased LPL activity could help to explain the increased clearance of postprandial TG. Accordingly, the DHA and EPA treatments reduced chylomicron diameter [38], indicating increased hydrolysis of TGs by LPL. In a follow-up analysis of the same study population, margination of TG-rich lipoproteins was measured as an estimation of lipoprotein activity [39]. The increased margination of TG-rich lipoproteins with DHA and EPA supplementation indicated that the treatment increased either the amount of endothelial bound LPL or the affinity of lipoproteins to LPL [39]. In a recent study, the effects of a hypocaloric diet plus 4 g daily OM3FA supplementation for 12 weeks on apoB48 kinetics after ingestion of a high-fat meal were investigated [40]. The clearance rate of apoB48 was increased by both the hypocaloric diet and the OM3FA treatment. Combined hypocaloric diet and OM3FA treatment as compared with hypocaloric diet alone reduced postprandial TG and apoB48 levels further, as well as reducing intestinal apoB48 production [40]. Thus, OM3FA treatment and weight reduction have an additive effect on chylomicron metabolism, postprandial TG and apoB48 levels.

In summary, OM3FA treatment increases the capacity for clearance of TG-rich lipoproteins, an effect observed in the postprandial but not in the fasting state, and DHA and EPA seem to have similar effects on postprandial TG levels [21, 38]. OM3FAs increase LPL activity, reflected by the higher clearance rate of TG-rich lipoproteins in the fed state but not in the fasted state, because the LPL capacity is not rate-limiting when TG levels are not very high. The reduced VLDL-TG production following OM3FA treatment is probably also of importance for enhanced chylomicron clearance, because that results in fewer VLDL particles competing with chylomicrons for TG hydrolysis by LPL. Finally, OM3FA treatment also seems to reduce the production of chylomicron particles in individuals receiving a hypocaloric diet.

### Lipoprotein lipase

As described in the previous section, OM3FA treatment increases individuals’ TG-rich lipoprotein clearance capacity, probably by increasing LPL activity at the endothelial surface. Several mechanisms might explain the increased LPL activity. One possibility is that OM3FAs increase LPL expression. In a randomized, double-blind, placebo-controlled study investigating the effects of 6 g fish oil daily for 6 weeks on men with atherogenic dyslipidemia, adipose tissue biopsies were taken 4–5 h post-breakfast [41]. Interestingly, LPL mRNA expression in adipose tissue increased in patients receiving fish oil supplementation. A potential mechanism for increased LPL mRNA expression is increased activation of the peroxisome proliferator-activated receptor response element of the *LPL* gene, which has been demonstrated using a transfection assay [42]. However, the major regulation of LPL activity takes place post-translationally. Detailed studies on post-translational regulation, such as transport and dimerization of LPL by OM3FAs, are generally lacking. Comparing pre-heparin and post-heparin LPL activity showed consistently increased pre-heparin activity, while post-heparin activity was unchanged by OM3FA treatment in most studies [38, 39, 43] but not all [41]. It is probable that neither pre-heparin nor post-heparin LPL activity accurately indicate the active endogenous pool of the protein on endothelial cells. Post-heparin LPL activity does not necessarily reflect physiologically active enzyme because all enzymes are activated by heparin. Pre-heparin activity is a measure of LPL released from endothelial cells, including endogenous plasma activators and inhibitors, and might therefore explain why pre- rather than post-heparin LPL activity is better associated with margination of TG-rich lipoproteins as an estimation of lipoprotein activity [39].

### Apolipoproteins E, CII and CIII

The activity of LPL is regulated by apolipoproteins on TG-rich lipoproteins. ApoCII is an endogenous activator, while apoCIII is an endogenous inhibitor of LPL. Treatment with OM3FAs decreases plasma apoCIII levels [7, 44]. EPA alone does not change total plasma levels of apoCIII or apoCII, but decreases apoCII and apoCIII in VLDL [45]. However, there are few studies investigating if EPA has a specific effect on plasma levels and distribution of apoCII and apoCIII on lipoprotein particles in comparison with DHA.

Apart from inhibiting LPL, apoCIII in VLDLs reduces clearance of VLDL particles by inhibiting the effect of apoE on receptor-mediated uptake [46]. The major cause of reduced plasma VLDL levels is reduced VLDL production; therefore, it is unlikely that this effect of apoCIII plays a major role in the regulation of VLDL levels following treatment with OM3FAs. Experimental studies show that apoCIII enhances hepatic assembly and secretion of VLDL, and human kinetic studies have shown an association between apoCIII and VLDL production [47]. However, it remains to be shown clinically that apoCIII influences VLDL production. ApoCIII is a predictor of CV disease and reduced apoCIII levels may have direct beneficial effects on the vessel wall, such as by reducing its retention of LDL particles (LDL-apoCIII) [48–50]. Therefore, reduction of plasma apoCIII by OM3FAs is potentially positive from the perspective of CV risk, but it is unclear to what extent it contributes mechanistically to fasting TG levels following treatment with OM3FAs.

### LDL levels, composition and particle size

In most studies investigating the effect of OM3FAs on LDL, there is no effect or a small increase in LDL-C levels. A consistent finding is an increase in mean LDL particle size, with a resulting shift towards those classified as large, rather than small, LDL particles [7, 45, 51–54]. Larger LDL particles may reduce CV risk because small dense LDL is associated with increased atherogenicity [55]. A reduction in large VLDL1 production [30] is the most likely explanation for the larger LDL particles after treatment with OM3FAs. Other potential OM3FA-related mechanisms that may explain the changes in LDL particle size are less likely and include effects on hepatic lipase or cholesteryl ester transfer protein (CETP) activity. Hepatic lipase activity is typically unchanged in controlled studies investigating the effects of OM3FAs on patients with dyslipidemia [38, 43, 52]. Inconsistent effects on CETP activity have been observed following treatment with OM3FAs: either no change [56], an increase [45] or a decrease [57] in activity.

#### Differential effects of DHA and EPA on LDL

Comparing EPA treatment with DHA treatment, or DHA treatment with OM3FA treatment, generally shows similar and small or no effects on LDL-C levels [18, 19, 23–26]. However, it is reported that treatment with DHA, but not EPA, increases LDL-C levels and LDL particle size [22, 54]. In contrast, one study showed no effect of DHA, but EPA treatment decreased TG levels and increased LDL-C levels in normolipidemic individuals [20].

One potential reason for the contradictory findings of OM3FA treatment on LDL-C levels could be an interaction between OM3FAs and *APOE* genotype. ApoE is mainly associated with LDL and VLDL particles in the circulation. It is a high-affinity ligand for the LDL receptor as well as for the LDL receptor-related receptor and thereby facilitates the hepatic uptake of LDL and VLDL particles. There are three major isoforms of apoE: apoE2, apoE3 and apoE4, which differ in the amino acids present at positions 112 and 158 of the protein. ApoE3 is the most common isoform (70–80%), while the prevalence of apoE4 is approximately 10–15% and that of apoE2 is approximately 5–10%.

ApoE4 is associated with a greater lipid response to dietary challenges [58]. Minihane et al. were the first to show that the effect of OM3FAs on LDL was dependent on *APOE* genotype [59]. Total cholesterol levels increased in *APOE4* carriers and, although not statistically significant, LDL-C levels tended to increase more in *APOE4* carriers than in *APOE2* or *APOE3* carriers following OM3FA treatment. However, small dense LDL levels decreased similarly in the different *APOE* genotypes [59]. The interaction between DHA and EPA response and *APOE* genotype (*APOE3*/*E3* vs *APOE*3/*E4*) was further investigated in a study comparing the effects of EPA-rich (3.3 g EPA) and DHA-rich (3.7 g DHA) oils, and a control oil, in a 3 × 4-week study separated by 10-week wash-out periods [5]. A differential response to DHA and EPA treatment was observed in *APOE4* carriers. DHA, but not EPA, treatment increased LDL-C in *APOE4* carriers, while LDL-C tended to decrease in *APOE3* carriers. A possible explanation for these findings is that VLDL2 particles from *APOE4* carriers reduce the uptake of LDL [5]. Thus, apoE4-containing VLDLs, in contrast to apoE3-containing VLDLs, compete with LDLs for uptake via the LDL receptor in hepatocytes, which could at least partly explain the higher LDL-C levels in *APOE4* carriers compared with *APOE3* carriers following DHA treatment. Another likely explanation for the increase in LDL-C in *APOE4* carriers is a greater conversion of VLDL to LDL, which has been shown to occur in *APOE4* carriers in kinetic studies [60]. Furthermore, it has been shown that *APOE4* carriers respond to fish oil supplementation with a larger increase in LPL mRNA and post-heparin plasma LPL activity than non-*APOE4* carriers [41]. This finding gives a plausible explanation for the greater conversion of VLDL to LDL in *APOE4* carriers than in *APOE3* carriers following OM3FA treatment.

#### Receptor-mediated LDL uptake

Other studies have investigated the uptake of LDL enriched in OM3FAs in hepatocytes. One study found no difference in the uptake of LDL enriched in OM3FAs compared with LDL particles not enriched in OM3FAs [51]. Another study found that LDL particles enriched in OM3FAs depressed both LDL receptor mRNA levels and activity to a larger extent than did control LDL [61]. Expression of LDL receptor mRNA in peripheral blood, used as a surrogate marker for expression in the liver, was not influenced by OM3FA treatment in normolipidemic individuals [51, 62], but decreased in hyperlipidemic individuals [62, 63]. Proprotein convertase subtilisin/kexin type 9 (PCSK9) is mainly produced by the liver and found in the circulation. It regulates LDL-C levels by promoting degradation of the LDL receptor. Treatment with a DHA-enriched oil [64] or an OM3FA formulation containing both DHA and EPA [65] reduced plasma levels of PCSK9, but not LDL-C. Therefore, PCSK9 is unlikely to be of major importance for LDL turnover following treatment with OM3FA.

To summarize the effects of OM3FAs on LDLs, a small increase in LDL-C levels in hyperlipidemic patients following treatment with OM3FAs could be explained by several, not mutually exclusive, mechanisms. Reduced VLDL production increases the rate of conversion of VLDL particles to IDL and LDL particles. Evidence from clinical studies suggests that reduced hepatic production of VLDL, especially VLDL1, reduces the TG load of LDL and therefore the LDL particles will contain more cholesterol (LDL-C) and become larger. In vitro results indicate that LDL enriched in OM3FAs could reduce LDL receptor activity to a larger extent than control LDL, which could contribute to increased LDL-C levels. Finally, there is a relationship between DHA and the apoE4 isoform, which results in increased production of LDL from VLDL as well as in reduced hepatic uptake of LDL via competition with apoE4-enriched VLDL2. Therefore, individual patients with the apoE4 variant could contribute to an overall increase in LDL-C levels in trials using OM3FA formulations containing DHA.

## Conclusions

In humans, EPA and DHA reduce plasma TG levels to a similar extent via two main mechanisms: 1) by reducing hepatic production of VLDL lipoproteins; and 2) by increasing postprandial LPL activity. Little is known regarding the specific cellular and biochemical effects of EPA and DHA that are responsible for the TG-lowering mechanisms in humans. Key findings of this review are summarized in Table 3.

**Table 3:** Summary of key findings

• There is no clear difference between DHA and EPA with respect to reducing fasting or postprandial TG levels.
• The major mechanism explaining reduced fasting serum TG associated with OM3FA treatment is reduced VLDL production, including a reduced number and size of VLDL particles. The reduced VLDL production results in a faster conversion of VLDL particles to IDL and LDL.
• OM3FA supplementation partly corrects the underlying disorder responsible for the atherogenic dyslipidemia in patients with type 2 diabetes by reducing hepatic production of VLDL1.
• Potential mechanisms for the inhibitory effect of OM3FAs on VLDL production include improved hepatic insulin sensitivity, reduced liver fat and increased whole-body fatty acid oxidation.
• There is a relationship between DHA and the apoE4 isoform of apoE, which results in an increased production of LDL from VLDL as well as in a reduced hepatic uptake of LDL via competition with apoE4-enriched VLDL2. Therefore, patients with the apoE4 variant could contribute to an overall increase in LDL-C in trials using OM3FA formulations containing DHA.
• OM3FAs increase LPL activity, likely by increased expression of the gene and reflected as increased pre-heparin LPL activity. Increased LPL activity can explain the higher clearance rate of TG-rich lipoproteins postprandially, but normally not in the fasted state because LPL capacity is not rate-limiting when TG levels are not high.
• Treatment with OM3FAs reduces plasma levels of PCSK9, but does not reduce LDL-C levels. Therefore, PCSK9 is unlikely to be of major importance for LDL-C levels following treatment with OM3FA.

*Apo* apolipoprotein, *DHA* docosahexaenoic acid, *EPA* eicosapentaenoic acid, *IDL* intermediate-density lipoprotein, *LDL* low-density lipoprotein, *LDL-C* low-density lipoprotein cholesterol, *LPL* lipoprotein lipase, *OM3FA* omega-3 fatty acid, *PCSK9* proprotein convertase subtilisin/kexin type 9, *TG* triglyceride, *VLDL* very-low-density lipoprotein

## Abbreviations

ALA: Alpha-linolenic acid
Apo: Apolipoprotein
CAD: Coronary artery disease
CE: Cholesterol esters
CV: Cardiovascular
DHA: Docosahexaenoic acid
EPA: Eicosapentaenoic acid
FFA: Free fatty acid
HDL: High-density lipoprotein
IDL: Intermediate-density lipoprotein
LDL: Low-density lipoprotein
LDL-C: Low-density lipoprotein cholesterol
LPL: Lipoprotein lipase
MeSH: Medical Subject Heading
OM3FA: Omega-3 fatty acid
OM6FA: Omega-6 fatty acid
PCSK9: Proprotein convertase subtilisin/kexin type 9
PL: Phospholipid
PPAR: Peroxisome proliferator-activated receptor
TG: Triglyceride
VLDL: Very-low-density lipoprotein
VLDL-C: Very-low-density lipoprotein cholesterol
VLDL-TG: Very-low-density lipoprotein triglyceride
